# Undeveloped Mammæ

**Published:** 1894-01

**Authors:** W. B. Mask

**Affiliations:** Flat Creek, La.


UNDEVELOPED MAMMÆ AND IRREGULAR MENSTRUATION WITH GENERAL DEBILITY.—I prescribed SANMETTO to my daughter in teaspoonful doses three times a day, who had been in a debilitated condition three years. The history of her case is as follows: Age seventeen years; menstruated at the age of fourteen years; her general health good up that time, but two and one half years ago I noticed a decline in her health. I also learned there was some irregularity in menstruation, and while in this debilitated condition she received quite a nervous shock owing to the death of her little brother. Since that time I have used various remedies to build her up, but her menstrual flow, as a rule, was scant, and the mammaries had not developed as my other daughters. She was troubled with a torpid liver, together with obstinate constipation. She complained of pain in right hypochondriac and left iliac regions. I could not discover any benefit from the use of the first bottle of SANMETTO, but hoping that it might prove beneficial, I continued its use. It affords me much pleasure now to report the result obtained from SANMETTO in the case. Since using the last bottle she has mended wonderfully indeed, and is to-day in better health than she has been for three or four years, has gained several pounds, ovarian neuralgia almost entirely gone, and mammaries developing nicely. W. B. MASK, M.D.

Flat Creek, La.

